# Ice-binding proteins that accumulate on different ice crystal planes produce distinct thermal hysteresis dynamics

**DOI:** 10.1098/rsif.2014.0526

**Published:** 2014-09-06

**Authors:** Ran Drori, Yeliz Celik, Peter L. Davies, Ido Braslavsky

**Affiliations:** 1 Institute of Biochemistry, Food Science and Nutrition, The Robert H. Smith Faculty of Agriculture, Food and Environment, The Hebrew University of Jerusalem, Rehovot, Israel; 2 Department of Physics and Astronomy, Ohio University, Athens, OH, USA; 3 Department of Biomedical and Molecular Sciences, Queen's University, Kingston, Ontario, Canada

**Keywords:** ice-binding, antifreeze proteins, thermal hysteresis

## Abstract

Ice-binding proteins that aid the survival of freeze-avoiding, cold-adapted organisms by inhibiting the growth of endogenous ice crystals are called antifreeze proteins (AFPs). The binding of AFPs to ice causes a separation between the melting point and the freezing point of the ice crystal (thermal hysteresis, TH). TH produced by hyperactive AFPs is an order of magnitude higher than that produced by a typical fish AFP. The basis for this difference in activity remains unclear. Here, we have compared the time dependence of TH activity for both hyperactive and moderately active AFPs using a custom-made nanolitre osmometer and a novel microfluidics system. We found that the TH activities of hyperactive AFPs were time-dependent, and that the TH activity of a moderate AFP was almost insensitive to time. Fluorescence microscopy measurement revealed that despite their higher TH activity, hyperactive AFPs from two insects (moth and beetle) took far longer to accumulate on the ice surface than did a moderately active fish AFP. An ice-binding protein from a bacterium that functions as an ice adhesin rather than as an antifreeze had intermediate TH properties. Nevertheless, the accumulation of this ice adhesion protein and the two hyperactive AFPs on the basal plane of ice is distinct and extensive, but not detectable for moderately active AFPs. Basal ice plane binding is the distinguishing feature of antifreeze hyperactivity, which is not strictly needed in fish that require only approximately 1°C of TH. Here, we found a correlation between the accumulation kinetics of the hyperactive AFP at the basal plane and the time sensitivity of the measured TH.

## 1. Introduction

Ice-binding proteins protect cold-environment organisms by limiting the growth of ice crystals within and/or outside their body fluids [1,2]. These proteins have been found in fish [3], plants [4], insects [5], fungi [6] and bacteria [7], and comprise antifreeze proteins (AFPs), ice recrystallization inhibition proteins and a newly discovered ice adhesion protein [8]. AFPs adsorb to the surfaces of ice crystals and lower the temperature at which an ice crystal grows, thereby creating a gap (thermal hysteresis or TH) between the melting point and the non-equilibrium freezing point in which ice growth is arrested. Although ice adhesion proteins show activities similar to those of AFPs, they serve as a means to adhere to ice and not necessarily to block its growth [8]. The accepted model explaining the inhibition of ice growth by AFPs is the adsorption–inhibition model. This model describes ice growth as occurring in the gaps between adsorbed AFPs [9]. This process increases the curvature of the ice surface between bound AFP molecules, thereby decreasing the radius of curvature from infinity to a finite magnitude. Such an increase in the surface curvature leads to a depression in the freezing point due to the Gibbs–Thomson effect, which states that the equilibrium melting point of a solid is related to the curvature of the solid surface and the interfacial energy [10,11]. Although the adsorption–inhibition model assumes irreversible binding of AFPs to ice, the irreversibility of this binding remains the subject of an ongoing debate in the AFP community [12–14].

Hyperactive AFPs found in insects and microorganisms (hypAFPs) are ten to a hundred times more active than moderate AFPs derived from fish and plants at equimolar concentration [15]. Moderate AFPs adsorb to prism and/or pyramidal planes of an ice crystal, whereas hypAFPs adsorb to these surfaces and the basal plane of an ice crystal [16,17]. The morphology of an ice crystal is influenced by the plane to which an AFP adsorbs. Moderate AFPs usually induce the formation of hexagonal bipyramidal crystals that form by growing, whereas hypAFPs usually produce crystals with hexagonal symmetry with curved surfaces that form during melting [17,18].

Although the dependence of TH activity on the AFP concentration has been extensively studied, the time dependence of this process remains unclear. A handful of experimental studies of the time-dependent TH activities of moderate AFPs have been reported [13,19–21], and a few recent studies of hypAFPs are available [12,22,23]; however, none of these studies has examined carefully the time-dependent TH activity of hypAFPs, nor have they elaborated on the mechanisms underlying AFP activity. Chapsky & Rubinsky found that the TH activity of a solution of AFP I from the winter flounder, *Pseudopleuronectes americanus*, could increase over time. The same researchers found that TH increased by a factor of 5 over time and reached a plateau 60–180 min after exposure of an ice crystal to the AFP I solution, depending on the concentration. They hypothesized that time-dependent TH resulted from the accumulation of AFP molecules on the ice surfaces or the rearrangement of adsorbed AFP molecules on the ice (or a combination of the two) [19]. Takamichi *et al.* observed the same phenomenon in a solution prepared from an AFPIII derived from Notched-fin eelpout. They found that the TH activity increased by a factor of 2.5 after allowing for an annealing time of 3 h [21]. The authors proposed a model for the observed TH effects that involved secondary binding of the AFP molecules to the convex ice front over time. A model for the kinetics of AFP ice growth inhibition was proposed by Kubota [13]. This model assumed the slow and reversible adsorption of AFPs to ice; thus, a period of time was needed to reach adsorption equilibrium. A working assumption of this model was that the AFPs reversibly adsorbed to ice. Wilson *et al.* [20] measured the accumulation of antifreeze glycoproteins (AFGPs) on the surfaces of ice crystals using ellipsometry techniques based on optical changes in the water–ice interface. They found that the signal from the interface changed over time until it reached a plateau at approximately 60 min. Their interpretation was that the change at the water–ice interface was caused by the adsorption of AFGP molecules to the ice crystal planes [20].

Various computational studies have suggested that AFPs form an ice-binding structure by constraining water molecules present at the ice-binding sites into a structural ordering that resembles ice [24,25]. Garnham *et al.* have found evidence for these protein-bound ice-like waters [26]. However, owing to the short lifetime of the ordered water structures on the AFP surfaces [27], it is likely that at any given time only a small proportion of the AFP molecules possess sufficient appropriately ordered water molecules to bind to ice. Higher AFP concentrations and longer exposure times should both increase the number of binding events between the AFP and ice.

Sander & Tkachenko developed a ‘kinetic pinning’ mathematical model that described the AFP molecules as obstacles that induced surface pinning on the growing ice surface [28]. The model described a reduction in the ice front propagation as a function of the AFP concentration. In their model, the growth velocity drops to zero as a function of concentration and once the growth stops the proteins continued to accumulate on the ice crystal surface. Ebbinghaus *et al*. [29] examined the dynamics of water molecules around the AFGP structure in solution using terahertz (THz) spectroscopy. They found that the AFGP hydration shell increased in size as the temperature was decreased and that the hydration shell has a depressed freezing point compared to bulk water. Ebbinghaus *et al.* suggested a mechanism by which AFGPs altered the dynamics of water molecules in the hydration shell in such a way that the solution freezing process was inhibited [29], thereby rejecting the adsorption–inhibition model. Recently, the same group measured the water dynamics around a hyperactive AFP (*Dendroides canadensis*, *Dc*AFP) and identified direct short-range interactions between the AFP and the ice surface, in addition to long-range ice–water interactions mediated by the AFP [14]. Solid-state NMR studies suggested that the ice-binding site of AFPIII became stripped of its hydration shell upon freezing, which enabled direct protein–ice contact [30]. Zepada *et al.* suggested that AFGPs interrupted the arrangement of the ice–water interface through kinetic effects rather than through mediating binding between the ice surface and water molecules. Their final conclusion was that AFGPs could not be accurately described using the Gibbs–Thomson model [31]. Knight & DeVries suggested that AFGPs adsorbed only to the non-basal planes of ice crystals, thereby influencing the crystal shape and minimizing the surface area of the unprotected basal plane. They offered a mechanism by which the rate of AFGP adsorption governed the depression of the non-equilibrium freezing point, and they proposed that this process was governed by the concentration of the proteins in the solution and not the time of exposure to AFPs [32]. These findings describe the kinetic effects of AFPs known at this time and illustrate the uncertainty and confusion in the field with regard to the mechanism of action of AFPs. The kinetic effects of the hyperactive AFPs as well as a unified understanding of the mechanisms at work in both the moderate AFP and hypAFP systems have not yet been established.

**Figure 1.**
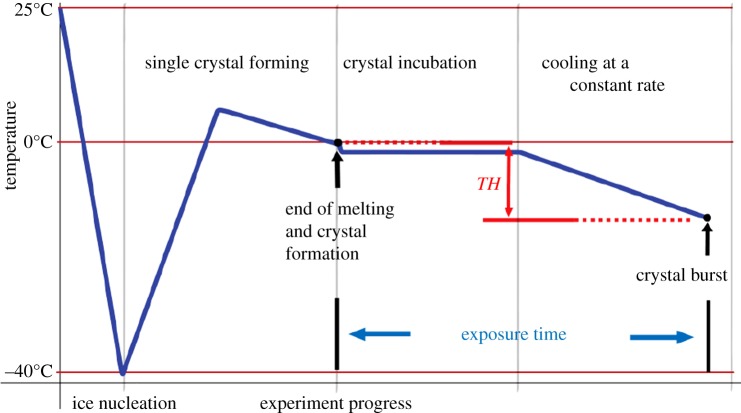
Stages of the measurement are indicated by text. Sample temperature in the nanolitre osmometer is represented by the bold line. TH is indicated by the double-headed vertical arrow. (Online version in colour.)

Standard methods for measuring TH activity (the difference between the melting point and the depressed temperature at which the ice crystal starts to grow) include the use of a nanolitre osmometer [22] in which a single crystal is formed and the melting point is identified with the aid of video microscopy and image analysis. The temperature of the system is then lowered at a fixed rate until the ice crystal undergoes a sudden burst of growth, which marks the non-equilibrium freezing point. The time separating the formation of a single crystal and the growth burst is defined as the ‘exposure time’ of the crystal (figure 1). During the exposure time, AFPs can adsorb to the ice crystal surface.

Our objectives in this work were to characterize the effects of the exposure time on the TH properties of various AFPs and to identify the distinctions between the kinetic mechanisms by which various AFPs function. We found that the TH activities of *Tenebrio molitor* AFP (*Tm*AFP) and spruce budworm AFP (sbwAFP) could be increased by a factor of 40 by increasing the exposure time. We examined the accumulation rates of both the moderate and hyperactive AFPs on the different crystal planes, as well as the TH activity.

The findings reported here suggest a new approach to measuring and enhancing TH activity. Appropriate kinetic models of AFP adsorption to ice crystal planes can improve our understanding of the mechanisms by which these proteins act and could lead to a unified description of AFP activity.

## 2. Material and methods

### 2.1. Thermal hysteresis activity measurements

We used a custom-designed nanolitre osmometer system that includes a 3040 Newport temperature controller (Irvine, CA, USA) and a previously described custom-made cooling stage [22,33]. The temperature of the cooling stage was controlled using a LabVIEW program developed in-house. An AVI file was generated by a CCD camera connected to a video frame grabber (IMAQ-PCI 1407, National Instruments, Austin, TX, USA) and was recorded to a PC. The images were collected at a rate of 30 frames s^−1^. TH measurements were as follows (figure 1): sub-microlitre volumes of AFP solutions were placed in immersion oil and were cooled to –30°C to generate freezing. The temperature was increased slightly above the melting point. The melting point (*T*_m_) was determined as described elsewhere [34]. The bulk ice was then melted until a single crystal 10–15 μm in size remained. These crystals were then incubated in the AFP solution for different lengths of time at a fixed temperature of typically 0.05°C below the *T*_m_. The temperature was subsequently decreased at a fixed rate (0.15°C per min by reducing the temperature by 0.01°C every 4 s) until the crystal underwent sudden, uncontrolled growth (burst). The time between the crystal formation (the end of melting) and the growth burst was defined as the crystal ‘exposure time’. The output AVI files were analysed after the experiments to accurately identify the melting point and the growth burst of the crystal [22]. The exposure times obtained from each experiment were binned to form clusters of similar exposure times. Each cluster comprised at least three experimental results, and the average TH of each cluster was plotted as a function of the exposure time (figures 3–6).

### 2.2. Proteins

Several proteins were tested in this study. Three hyperactive GFP-tagged AFPs were produced in *Escherichia coli*: *Tm*AFP-GFP [35] was stored in a solution containing 20 mM ammonium bicarbonate buffered at pH 8; *Mp*AFP-GFP from *Marinomonas primoryensis* [36] was stored in 20 mM CaCl_2_, 25 mM Tris–HCl (pH 8); and isoform 501 of sbwAFP-GFP [17] was stored in 20 mM Tris–HCl (pH 8). The GFP-tagged moderately active AFP derived from ocean pout, AFPIII-GFP [33] was stored in 100 mM ammonium bicarbonate buffered at pH 8.

### 2.3. Microfluidic device fabrication and microfluidic experiments

**Figure 2.**
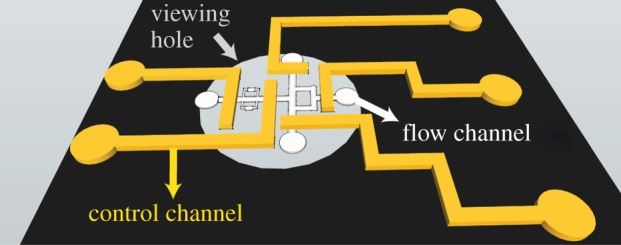
Microfluidic chip design. The control layer (yellow) was used as pneumatic valves and was fabricated on top of the flow layer (white), which was designed to fit the viewing hole of the cooled stage (5 mm in diameter). The crystals were formed in the small side compartments in the flow layer. During AFP accumulation, these compartments were isolated from any flow using the pneumatic valves.

Previously, we developed and used microfluidic devices to understand the effects of free unbound AFPs on ice crystal growth and the binding mechanism of the AFPs to ice [12]. With the assistance of Gerber's lab in Bar Ilan University, we further improved the devices to allow for the exchange of solutions in a rapid and controllable manner, using a two-layer fabrication technique that includes one layer for liquid handling and one layer that provided actuation of a set of valves. These devices were used to obtain ice crystals in which only the crystal surfaces were bound by the AFPs. This binding mode was achieved by growing the ice in a solution that did not contain AFPs prior to the introduction of the AFPs. This device was fabricated using a standard protocol provided in [37,38], and further described in the electronic supplementary material. The microfluidic device consisted of two aligned layers—a flow layer that included the fluid (figure 2, white), and a control layer (yellow) that included the pneumatic valves. The completed device was placed on a cold stage controlled using a temperature controller (Model 3150, Newport, Irvine, CA, USA) that was governed by a LabVIEW program described in [12]. The cold stage was mounted on a fluorescence microscope (Ti Eclipse, Nikon, Japan) outfitted with a sCMOS camera (Neo 5.5 sCMOS, Andor, UK) used for visualization.

AFP-free buffer solution in the microfluidic channel spontaneously nucleated to form a bulk ice crystal at approximately –20°C. The bulk ice crystal was subsequently warmed to a temperature near the equilibrium melting temperature to form a single crystal. A 980 nm, 500 mW, diode laser (Wuhan Laserlands Laser Equipment Co., ltd, China) was used to heat and melt any unwanted ice to help obtain the single crystal in the designated cell. After ice formation, AFP solution was injected to the flow line using a glass syringe. By this method, no AFP was present in the core of the ice and the AFP molecules adsorbed to the crystals only at the surface (see the electronic supplementary material, figure S1).

## 3. Results

### 3.1. The thermal hysteresis activity of *Tenebrio molitor* antifreeze protein increased by a factor of 10 over a prolonged exposure time

**Figure 3.**
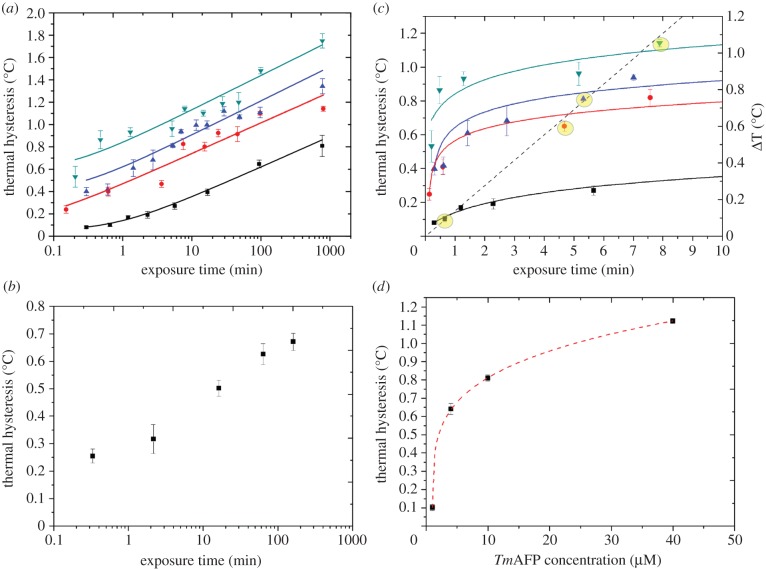
Effect on TH of the exposure time between ice and *Tm*AFP. Solutions of different concentrations were tested for TH as a function of *Tm*AFP exposure time: 1 µM (black squares), 4 µM (red circles), 10 µM (blue triangles) and 40 µM (green inverted triangles). (*a*) Plots of TH versus exposure time on a log scale for the different *Tm*AFP concentrations. TH values were the average of 3–10 measurements for each data point, with the variability indicated by the vertical error bars. (*b*) The untagged *Tm*AFP were tested for time dependence at a concentration of 10 µM. TH values were the average of 3–5 measurements for each data point, with the variability indicated by the vertical error bars. (*c*) Re-plotted data from (*a*) where the closest data points to the intersection of a 0.15°C min^−1^ constant cooling rate (dashed line) are highlighted in yellow. (*d*) The highlighted data points from (*b*) are plotted to examine the dependence of TH on the concentration of *Tm*AFP. The curved lines in (*a*,*d*) were fitted using Origin software.

*Tm*AFP is a 9 kDa protein with eight disulfide bonds [39]. We used GFP labelled *Tm*AFP. The positioning of the GFP tag in the fusion protein did not interfere with the ability of the proteins during binding to ice and have actually enhanced the TH activity as a result of the larger protein size (36 kDa) [33,40] (figure 3*a,b*). The addition of 27 kDa to the AFP may, however, have slowed down the diffusion rate and influenced the adsorption rate. We tested the significance of the mass effects by comparing the dependence of TH on incubation time for the *Tm*AFP and *Tm*AFP-GFP proteins under several conditions. Indistinguishable kinetic behaviours were observed (figure 3*a,b*).

The kinetics of the TH activity were measured using a custom-made nanolitre osmometer. The procedure is illustrated schematically in figure 1 and described in Material and methods and in [22]. Measurements of the TH activity as a function of the exposure time revealed that the TH activity in the *Tm*AFP solution increased with the exposure time for all solution concentrations measured (figure 3*a*). The TH activity increased by factors of between 10 (for the 1 µM solution) and 3 (for the 40 µM solution). A long exposure time (12 h) at a very low *Tm*AFP concentration (1 µM) increased the TH activity to a level comparable to that obtained from incubating an ice crystal in a 10 µM solution for a much shorter period of time (10 min).

Interestingly, the TH value did not reach a plateau at long exposure times, where 13 h was the longest time the TH value was tested. The TH obtained from the *Tm*AFP solutions obviously depended on the concentration (figure 3*a*). A plot of the TH values obtained from the four *Tm*AFP concentrations as a function of the exposure time followed an approximately hyperbolic relationship (figure 3*c*). The exposure time was calculated as the sum of the incubation time and the cooling time until bursting. Higher TH values were obtained with extended crystal incubation times (figure 1). In many experiments published previously in the literature, a constant cooling rate without an incubation time was used [9,15]. Thus, the exposure time was the same as the cooling time. We note that such a cooling scheme intersects with the four concentration curves shown in figure 3*c* for a cooling rate of 0.15°C min^−1^. Thus, it was possible to directly plot the effects of the *Tm*AFP concentration on the TH under such cooling conditions, as shown in figure 3*d*. The hyperbolic relationship was similar to other plots of the TH versus the AFP concentration [9,15]; however, the latter represent only part of the TH concentration dependence, without the full impact of the time dependence, as shown in figure 3*c*.

### 3.2. Each antifreeze protein displayed different kinetics

**Figure 4.**
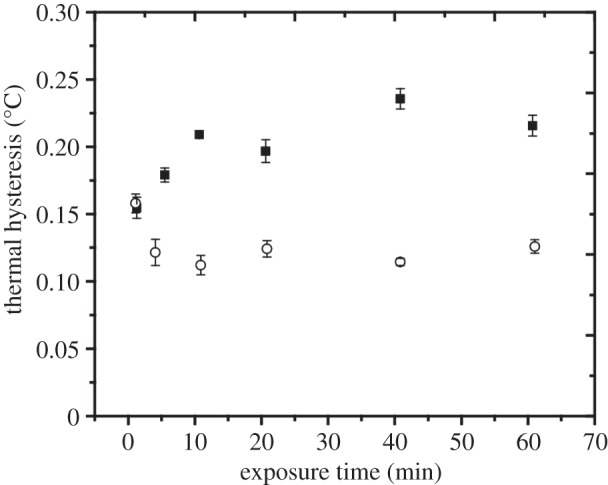
Sensitivity of TH to the AFPIII exposure time. The TH activity of a 40 µM AFPIII solution was examined as a function of ice crystal exposure time. TH values are the average of at least three measurements taken at 0.02°C below *T*_m_ (open circles) and 0.1°C below *T*_m_ (filled squares), with the variability indicated by the vertical error bars.

The kinetics of the TH effects were further examined by conducting exposure time-dependent TH measurements using different AFPs. Takamichi *et al.* showed that the TH activity of moderately active fish AFPIII from notched-fin eelpout was influenced by the exposure time [21]. These authors additionally found that the temperature at which the crystal was incubated prior to the TH measurement (during ice crystal stasis) affected the TH measurements. A solution incubated under highly supercooled conditions (at 0.25°C below the melting point) yielded a TH activity that was a factor of 1.7 times the TH activity obtained by incubating the crystal under less supercooled conditions (at 0.05°C below the melting point). This experiment was repeated here using the AFPIII from ocean pout (at a concentration of 40 µM, which corresponded to the maximum concentration of *Tm*AFP tested here) and under two supercooling conditions (incubation at 0.02°C or 0.1°C below the melting point). The results (figure 4) qualitatively agreed with previous findings [21]. A short exposure time (a few seconds) under highly supercooling conditions produced a slightly lower TH activity than a longer exposure time. Under a small degree of supercooling (incubation at 0.02°C below the melting point), the TH activity was unaffected by the exposure time.

**Figure 5.**
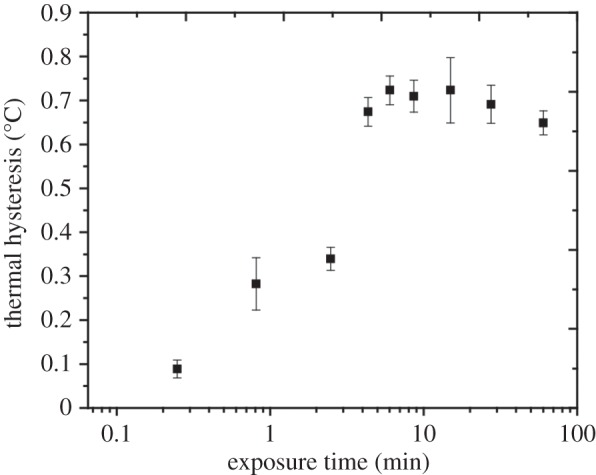
Sensitivity of TH activity to *Mp*AFP exposure time. The TH activity of a 2.4 µM *Mp*AFP solution was plotted against the log of the exposure time. TH values (black squares) are the average of at least three measurements with the variability indicated by the vertical error bars. (Adapted with permission from [22].)

The hyperactive AFP derived from the Antarctic bacterium *Marinomonas primoryensis* (*Mp*AFP) is a 38 kDa protein and is somewhat larger than most AFPs (3–16 kDa). The adsorption kinetics of *Mp*AFP to ice were distinct from those of AFPIII, although they were closer to the kinetics of AFPIII than to the kinetics of *Tm*AFP. The TH activity was found to depend on the exposure time only at low concentrations (2.4 µM) of *Mp*AFP-GFP (figure 5) [22]. At higher concentration (4.2 µM), the TH activity increased significantly over very short exposure times and did not increase at all over time at much higher concentrations (8–55 µM, data not shown). After a 4 min exposure time, the TH activity reached a plateau. This plateau was not observed among the *Tm*AFP solutions, even after 13 h, but was obtained from the AFPIII solutions after a few minutes. At low concentrations, the kinetics of *Mp*AFP were similar to those obtained from *Tm*AFP (figure 3). After an exposure time of a few minutes, the TH activity remained unchanged. Unlike AFPIII, the degree of the solution supercooling during the exposure and TH measurements did not affect the TH activity of the *Mp*AFP solutions.

**Figure 6.**
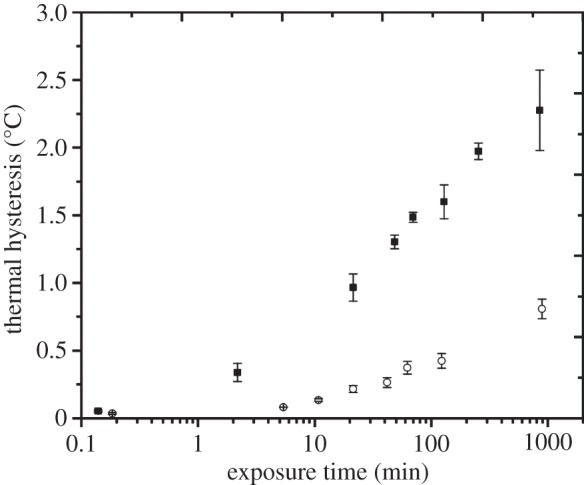
Sensitivity of the TH activity to sbwAFP exposure time. TH activity of 4 µM (open circles) and 8 µM (filled squares) sbwAFP solutions plotted against the log of the exposure time. TH values are the average of at least three measurements with the variability indicated by the vertical error bars.

A third hyperactive AFP, sbwAFP from the spruce budworm moth, *Choristoneura fumiferana*, was tested. This protein is 12 kDa in mass and contains TXT motifs (Thr-X-Thr, where X is any amino acid) on its ice-binding site, similar to those found in *Tm*AFP and its orthologue from *Dc*AFP [41,42]. As with *Tm*AFP, The TH of sbwAFP depended profoundly on the exposure time (figure 6). Very short exposure times to the sbwAFP solutions (several seconds) did not produce TH activity. A longer exposure time of a few minutes in the presence of higher protein concentrations (8 μM) yielded a TH of 1°C, and the TH continued to increase to 2°C as the exposure time increased (figure 6). The lowest concentration solution tested (4 μM) also showed a remarkable TH enhancement over time (figure 6). These results suggested that adsorption rate of sbwAFP was smaller than the adsorption rate of *Tm*AFP (i.e. more time was needed for sbwAFP to depress the freezing point) and was extremely slow compared with adsorption rate of AFPIII. The TH activity increased by a factor of 40 as the sbwAFP exposure time was increased from 8 s to 850 min. This TH ratio increase value was the highest value yet measured among the AFPs tested thus far (figure 6). We hypothesize that the AFPs accumulated on the ice surface over time, and this accumulation increased the measured TH activity.

### 3.3. Direct measurement of the antifreeze protein accumulation rates using fluorescence intensity measurements

The accumulation of AFP molecules on the ice surfaces was measured by imaging the ice crystals in the presence of AFP molecules fused to GFP markers, AFP-GFP. Microfluidic techniques were used to prepare a large ice crystal in an AFP-free solution. An AFP-GFP solution was then injected into the microfluidic channel, and the large crystal was partially melted. Once melting had ceased, the AFP-GFP molecules adsorbed to the ice crystal surface under constant temperature conditions (approx. 0.05°C below the melting point of the crystal). The outer layer of the crystal then became covered with AFP-GFPs, and no AFP-GFPs were observed within the crystal. Note that if the ice crystals had been initially nucleated in solutions containing AFP-GFP, the crystals that formed would have included AFP-GFPs within the ice, and not only on the crystal surfaces. This procedure was performed for both the AFPIII-GFP and *Tm*AFP-GFP. There is a difference between the two AFP types: ice in *Tm*AFP-GFP solution is shaped during melting, while the crystals in AFPIII-GFP are shaped within the hysteresis gap while growing from an initial ice seed in the presence of AFPIII-GFP. Consequently, the ice grown from an AFP-free seed in the presence of AFPIII-GFP does not contain fluorescence within the ice because AFPIII-GFP does not adhere to the basal plane and therefore will not get incorporated into the ice during formation of the bipyramidal tips by growth along the *c*-axis. For example, a bright core was observed in crystals formed in the presence of AFPIII-GFP (electronic supplementary material, figure S1*a* and fig. 3*b* in [33]). The formation of ice crystals from AFP-GFP-free solutions prior to exposing the crystals to an AFP-GFP solution is a unique feature of the experiments conducted here using a microfluidic apparatus. This procedure resulted in crystals that were bound by AFP-GFP only on their surfaces (electronic supplementary material, figure S1*b*).

**Figure 7.**
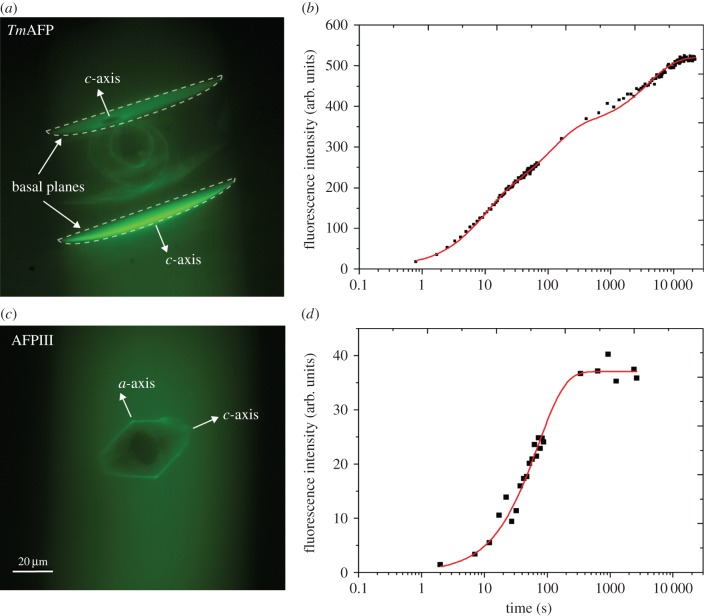
Accumulation of *Tm*AFP and AFPIII on different ice crystal planes. (*a*,*b*) *Tm*AFP and (*c*,*d*) AFPIII. (*a*,*c*) Ice crystals with surface-adsorbed AFP-GFP. Crystal axes are indicated by white arrows, and their dimensions are indicated by the scale bar. In the upper image (*a*), the two concentric lemon-shaped crystals are the seed for the observed basal plane. See the electronic supplementary material, movie S5, for the development of the basal planes from these seeds. The seed crystals are slightly tilted thus the projection of the basal planes that emerge from them is seen in the optical observation direction that is perpendicular to the microfluidic channel. Note that the apparent lens shape of the basal plane (marked with dotted lines) is caused by the round shape of the microfluidic channels (see the electronic supplementary material, figure S3). The scale bar is the same for both images. (*b*,*d*) Measurement of the fluorescence intensity of surface-adsorbed AFP-GFPs over time on the basal planes (black squares, *Tm*AFP) and prism plane (black squares, AFPIII). The red lines were plotted using the equations presented in the results section for each AFP.

The fluorescence signal was measured and extracted using the Nis-Elements program (Nikon, Japan). An in-house Matlab program was used to analyse the fluorescence signals and extract a maximum from each line profile of the fluorescence intensity perpendicular to the crystal plane that crossed the ice–solution interface. This procedure was used to measure the fluorescence signal from the ice surface over time in the microfluidic channel. The intensity was interpreted as indicating the amount of bound AFP present on the ice surface and was used to monitor the binding of both AFPIII-GFP and *Tm*AFP-GFP. Figure 7 plots the accumulation of AFP-GFPs as a function of time onto different planes of an ice crystal. The hyperactive AFP (*Tm*AFP, figure 7*a*,*b*) displayed continuous accumulation of *Tm*AFP-GFP molecules on the basal plane over an exposure time of 4 h. We did not observe further accumulation during an additional 1.5 h of exposure time suggesting that any subsequent change in fluorescence was too small to detect using our instrumentation. The accumulation on the basal plane revealed that the adsorption rate (the time derivative of the fluorescence intensity) was high within the first few minutes after ice growth had ceased. After approximately 1000 s, the adsorption rate slowed and nulled after approximately 4 h. The accumulation kinetics measurements over periods of at least 1 h were repeated six times. Accumulation experiments over shorter periods of time were repeated a greater number of times (more than 200 experiments). The accumulation experiments performed using AFPIII (figure 7*c*,*d*) showed that the accumulation of AFPIII-GFP at the prism plane reached saturation very rapidly (within 6 min) compared to *Tm*AFP-GFP (within 4 h). The solid line in figure 7*d* followed the equation
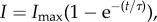
where *I* is the fluorescence intensity, *I*_max_ is the maximum fluorescence intensity parameter, *t* is the time from the ice formation and *τ* = 70 s is a time constant parameter that fit the data. Our experiments revealed that 6 s ≥ *τ* ≥ 90 s for AFPIII-GFP depending on the solution concentration. We found a linear correlation between the inverted τ and the concentration. Assuming that 1/*τ = K*_on_*C*, where *K*_on_ is the adsorption constant and *C* is the AFP concentration in the solution, we found that *K*_on_ = 0.008 ± 0.001 µM^−1^ s^−1^ (electronic supplementary material, figure S2). Nevertheless, single exponent formula did not fit the data for *Tm*AFP-GFP for any parameter set. A model that included three exponents to describe the process of *Tm*AFP accumulation, however, provided a good description of the data
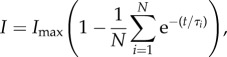
where *N* = 3 and *τ*_*i*_ = (9,110 and 4050)s, respectively.

The lack of binding between the moderate AFPs and the basal plane has been discussed previously [15] and has been shown qualitatively using AFPIII-GFP [17]. Here, we have directly and quantitatively measured this effect, verifying that this AFP does not bind to the basal plane (electronic supplementary material, figure S4). In summary, the fluorescence experiments reported here suggest that the adsorption and accumulation of AFP-GFPs onto ice surfaces is a two-step process. The first step (initial adsorption) is rapid and causes ice growth to cease, whereas the second step (accumulation) is slow. The adsorption of the hyperactive AFP was much slower and mainly occurred at the basal plane, whereas the moderate AFP reached a saturated adsorption state quickly, and no basal plane accumulation was observed. Electronic supplementary material, movie S5, is the experimental record of the formation of the crystal and the fluorescence signal over time that is shown in figure 7*a*,*b*.

## 4. Discussion

This study examined the influence of the exposure time, at a temperature slightly below the *T*_m_, on the hysteresis activities of several AFPs. This influence was then found to be correlated with the accumulation of the tagged AFPs on the surfaces of the ice crystals. Our study of moderate and hyperactive AFPs revealed very different TH dynamics. This study showed that the TH of hyperactive AFPs increased with longer AFP exposure times. Although the TH activities of the *Tm*AFP and sbwAFP solutions were sensitive to the exposure time (the TH activity increased by a factor of up to 40 as the exposure time was increased from a few seconds to several hours), the TH activity of the AFPIII solution was relatively insensitive to the exposure time (the TH activity varied by only a factor of 1.5 in an hour). The TH activity of *Mp*AFP, a bacterial hyperactive AFP, was only sensitive to the exposure time at low concentrations (2.4 µM). Over short exposure times (a few seconds), all hyperactive AFPs showed very low TH activity. The differences between the kinetics suggested that *Tm*AFP and sbwAFP bound to ice at a much slower rate than *Mp*AFP, which bound slower than the moderately active AFP. The rapid adsorption of AFPIII to the ice crystal could explain the observed TH activity. Evidence for rapid adsorption was obtained from fluorescence microscopy measurements during ice formation in the presence of AFPIII-GFP. Significant amounts of the measured fluorescence signal on fast growing ice was observed the instant the ice growth halted. In AFPIII accumulation experiments, in which the fluorescence intensity was measured after the crystal had melted to a smaller size and then shaped slowly to a bipyramidal structure (electronic supplementary material, movie S6), we measured single exponent intensity growth. The fast increase in the fluorescence signal from the surface of the fast growing crystals might be due to high local AFP concentration that developed during growth. Alternatively, this fast kinetics can attribute to modified ice surface in comparison to the gentle growth within the TH gap. We note that in the presence of the moderate AFP, the ice grew along the *c*-axis, and the ice surface developed within the hysteresis gap. This behaviour contrasts with ice crystal shaping in the presence of hypAFP that occurs during ice melting and not within the TH gap [18]. The rapid adsorption of AFPIII to the ice reduced the sensitivity of the TH to the cooling rate and could explain the small variations in the TH measurements obtained in the presence of the moderate AFPs, which contrasted with the large variations seen with hypAFPs, as reported by Scotter *et al*. [15]. Note that for our experiments conducted in a microfluidic device, ice crystals were formed in water without AFP, after which a solution containing AFP molecules was added to the experimental chamber. Prior to designing this microfluidic apparatus, it was not possible to form a single ice crystal without a core of fluorescent protein in the ice (electronic supplementary material, figure S1*b*). This unique feature both enhanced the ability to measure fluorescence on the surfaces and indicated that the AFP trapped inside of an ice crystal did not influence the TH.

It was not surprising that the two insect AFPs displayed similar TH dynamics, because these two proteins have the same function of protecting their hosts from freezing in sub-zero temperatures. The proteins also have very similar ice-binding sites within the protein structure that comprise tandemly arrayed TXT motifs. *Mp*AFP, on the other hand, includes the ice-binding domain of an ice adhesin is thought to be involved in docking its host bacterium to an ice crystal [8]. Although it is indeed a hypAFP, it has not evolved to be efficient at TH. Its TH activity curve as a function of AFP concentration differed from that of typical AFPs [36]. *Mp*AFP showed an almost sigmoidal increase in TH activity, but then rapidly approached a plateau value slightly greater than 2°C. By contrast, the insect AFPs approached TH values of 5–6°C at modest AFP concentrations [15]. In nature, freeze-avoiding insects, such as the *Tenebrio molitor* beetle and spruce budworm, can endure much lower temperatures (–20°C) than are indicated by the TH measured in the nanolitre osmometer [43]. This anomaly may be partially resolved by the fact that insects also produce colligative cryoprotectant agents, such as glycerol, to assist with freezing point lowering [43]. Another contributing factor is the effect of the crystal size on TH activity, as discussed by Zachariassen & Husby [44]. Crystals formed in the nanolitre osmometer (approx. 15 µm) are larger than those that form inside an insect [44]. Our findings may suggest yet another mechanism for resolving the differences between the *in vitro* TH measurements and the survival temperatures of the insects. Long exposure times to tiny ice crystals, as is allowed in the haemolymph of an insect, can increase the apparent TH in the insect beyond the values that are typically measured in the nanolitre osmometer.

The binding kinetics of the AFPs to ice were characterized using fluorescence microscopy techniques to measure the accumulation of the AFPs on the different ice crystal planes. Takamichi *et al.* [21] measured the accumulation of AFPIII fused to GFP and suggested that AFPs may progressively bind to ice over a few hours. Their measurement did not differentiate between the accumulation kinetics at different crystal planes, and a hyperactive AFP was not tested. Ellipsometry experiments performed by Wilson *et al.* [20] estimated the accumulation of AFGPs on ice surfaces. They found that the AFGPs accumulated on the ice surfaces over time, and a plateau in accumulation was observed after approximately 60 min at the prism plane. They also reported that the AFGPs did not appear to desorb from the ice surfaces [20]. Their results have met with skepticism due to certain inaccuracies in the measurements that discredited surface accumulation over time [45].

Our direct measurements of the AFP accumulation rates revealed that the initial adsorption of the AFPs to ice (over a few seconds) was very fast, and this process was similar for the AFPIII and *Tm*AFP. Subsequently, the hyperactive *Tm*AFP accumulated progressively on the basal plane over a few hours. The following section discusses these results provides an interpretation to place the results into context.

Several models have been proposed to describe the kinetics of AFP adsorption to ice [13,28]. For example, Kubota's work described the slow adsorption and desorption of AFPs, yielding a function that correlated the surface coverage with time as 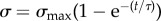, where *σ* is the surface coverage, *σ*_max_ is the maximum possible coverage, *t* is the time from the ice formation and *τ* is the time constant for the accumulation. This equation is a modified version of the original model that takes into account the incubation time. Indeed, our measurements of the AFPIII-GFP fluorescence intensity over time were consistent with this model, as shown in figure 7*d*. Knight & DeVries suggested that the inhibition of ice growth by those AFPs that could not bind to the basal plane (moderate AFPs) was not time-dependent and was instead a function of the AFP concentration and the degree of supercooling [32]. Thus, considering that AFPIII could not bind to the basal plane, the accumulation on the prism plane did not contribute to the TH activity. Indeed, AFPIII adsorbed to the prism plane rapidly (*K*_on_ = 0.008 ± 0.001 µM^−1^ s^−1^), and the TH was found to be insensitive (relative to the behaviour of hypAFP) to the exposure time. The modest time dependence occurred on a timescale that exceeded the accumulation kinetics of the fluorescence signal. The measured *K*_on_ value presented here is the first experimental report of the adsorption constant of AFP molecules to ice.

On the other hand, the hyperactive *Tm*AFP accumulated on the basal plane in a slow and progressive manner. Our measurements of the accumulation of *Tm*AFP did not reveal any particular timescale (*τ*). A multi-exponential function with at least three time constants was needed to describe the fluorescence data. Processes that must be modelled across many timescales or through the use of a stretched exponential are common in annealing processes, for example, in glass ageing [46]. The Weibull distribution [47] describes systems in which the durability of a bacteria population changes over time, and the time to decimation is not constant. In our system, multiple timescales could indicate that the adsorption of *Tm*AFP-GFP to ice was influenced by both the number of binding sites and by another process, such as the suitability of the available binding sites to interactions with AFP. The secondary binding process proposed by Takamichi *et al.* [21] provides a possible example of such a process. Garnham *et al.* [26] suggested that the pattern of bound water may also contribute to these dynamics. Several subpopulations of the AFP-bound water molecules may potentially fit different residual binding sites on the ice. The basal plane binding could explain the enhanced TH activity of the *Tm*AFP and sbwAFP over long periods of time, as was observed in our TH measurements. Accordingly, the ability of the hypAFPs to bind to the basal plane and to inhibit ice growth on this plane contributed most significantly to the time-dependent TH activity.

The interpretations of our results may be summarized in terms of two kinetic processes that contribute to TH. First, the AFPs blocked ice crystal growth in a concentration-dependent manner, as described by Sander *et al*. [28] and Knight & DeVries [32]. According to their theory, the velocity of an advancing ice front reduces to zero at certain AFP concentrations, depending on the degree of supercooling. Once the AFPs are adsorbed onto an ice crystal surface and the ice growth has been arrested, the second kinetic process of protein accumulation governs the TH. Sander suggested this possibility in previous publications [28]. We found that the hyperactive and moderate AFPs differed in their abilities to further inhibit crystal growth. HypAFPs accumulated over time, particularly on the basal plane, and protected the crystal from prematurely ending its TH. Moderate AFPs cannot bind to the basal plane and, for this reason, do not display kinetics that depend significantly on the incubation time. As mentioned above, Takamichi *et al.* [21] explained their measurements of TH enhancement over time by hypothesizing a ‘secondary binding’ conformation of AFPs. This explanation would suggest, however, that the moderate AFPs that bound in such configurations onto the prism plane only did not directly affect the TH activity. Alternatively, the TH enhancement over time by the moderate AFPs may be explained by a slow decrease in the basal plane surface area as was mentioned by Knight & DeVries [32]. One supporting piece of evidence for this claim is that when a crystal was incubated at a higher degree of supercooling, the extent to which the incubation time enhanced the TH increased. Thus, a bipyramidal crystal that is incubated at a high degree of supercooling, gradually grows in the *c*-direction and consequently decreases the basal plane surface area which leads to higher TH activities.

Our experiments using fluorescence microscopy techniques in conjunction with microfluidic devices demonstrated the binding of AFPs to ice crystals during the active exchange of solutions around a crystal. This study revealed the complex dynamics of protein attachment to the ice crystals and the time-dependent effects of protein attachment on the TH activity. The sensitivity of TH to the exposure time highlights the necessity of reporting the exposure time parameter along with any reported AFP TH activity. This is especially important for the TH activities of proteins that are highly sensitive to the exposure time, such as sbwAFP and *Tm*AFP. The relationship between the number of AFP molecules accumulated onto an ice surface and the measured TH will be investigated further in future studies.

## Supplementary Material

Electronic supplementary material
