# Supplementary material for: Ice-binding proteins that accumulate on different ice crystal planes produce distinct thermal hysteresis dynamics
Source: J R Soc Interface. 2014 Sep 6;11(98):20140526. doi: 10.1098/rsif.2014.0526 (PMC4233703; doi:10.1098/rsif.2014.0526)
Supplement: Electronic supplementary material [file rsif20140526supp1.doc]

Ice-Binding Proteins that Accumulate on Different Ice Crystal Planes Produces Distinct Thermal Hysteresis Dynamics

Ran Drori^1^, Yeliz Celik^2^, Peter L. Davies^3^ and Ido Braslavsky^1,2^

^1^Institute of Biochemistry, Food Science and Nutrition, The Robert H. Smith Faculty of Agriculture, Food and Environment , The Hebrew University of Jerusalem, Rehovot, Israel,

^2^Department of Physics and Astronomy, Ohio University, Athens, OH, USA,

^3^Department of Biomedical and Molecular Sciences, Queen’s University, Kingston, ON, Canada.

Supporting Information

**Mold fabrication**

The microfluidic device consisted of two aligned layers – a flow layer that included the fluid (Fig. 2, white), and a control layer (yellow) that included the pneumatic valves. The standard protocol of was modified as follows. Flow molds were fabricated on 3-inch diameter silicon wafers (Silicon Quest International, USA) that had been coated with hexamethyldisilazane in a vapor bath for 10 min. The wafers were spin-coated with the positive photoresist SPR 220-7 (Shipley) at 1500 rpm for 60 sec in a spin coater apparatus (Laurell, USA), resulting in a substrate height of 12–14 μm. The molds were then baked at 105 °C for 6 min, followed by a 60-s I-line exposure under an MA6 contact mask aligner (Karl Suss, Germany). Next, the molds were baked at 110 °C for 10 min, developed using the MF319 developer (ROHM and HAAS, USA), and washed with H2O. Finally, the molds were annealed under a steady temperature ramp (70–200 °C, 10 °C/h) over 15 h. Control molds were fabricated on 3-inch silicon wafers by spin-coating the negative photoresist SU-8 2025 (MicroChem, USA) initially at 500 rpm for 10 s, then at 3000 rpm for 60 s to yield a substrate height of 16–20 m. The molds were baked at 65 °C for 2 min and 95 °C for 5 min. The wafers were then exposed for 6 s under the mask aligner and post-exposure baked at 65 °C for 1 min and 95 °C for 3 min. The wafers were then developed in PGMEA developer (KMG) for 4.5 min and washed with isopropanol.

**Device fabrication**

The device fabrication procedure was adopted with modifications from . The molds prepared as described above were exposed to chlorotrimethylsilane (TMCS, Aldrich, USA) vapor for 10 min. The microfluidic devices were fabricated using a casting silicone elastomer, polydimethylsiloxane (PDMS, SYLGARD 184, Dow Corning, USA). A mixture of the silicone-based elastomer and curing agent was prepared in two ratios of 5:1 or 20:1, for the control and flow molds, respectively. The control layer was degassed and baked for 30 min at 80 °C. The flow layer was initially spin-coated (Laurell, USA) at 1800 rpm for 60 s and baked at 80 °C for 30 min. The control layer was separated from its mold, and control channel access holes were punched. The flow and control layers were then aligned and baked for 1.5 h at 80 °C. The two-layer device (chip) was peeled from the flow mold, and flow channels access holes were punched. The mold and chip fabrication steps were performed by Doron Gerber's lab at Bar Ilan University. The chips were bonded to a glass cover slip after treating with oxygen plasma cleaner (Harrick Plasma Cleaner, Model: PDC-32G, NY, USA). After device fabrication, a BSA solution (1%) was injected into the channels and incubated for 20 min to precoat non-specific protein binding sites and prevent GFP-tagged molecules from attaching to the PDMS. The control channels were filled with 50% (w/w) Ethylene glycol in DDW or Immersion oil Type FF (Cargille Laboratories Inc, Cedar Grove, NJ, USA) to enable rapid closing/opening of the valves, and to ensure that no freezing of the solution in the control channels will occur.


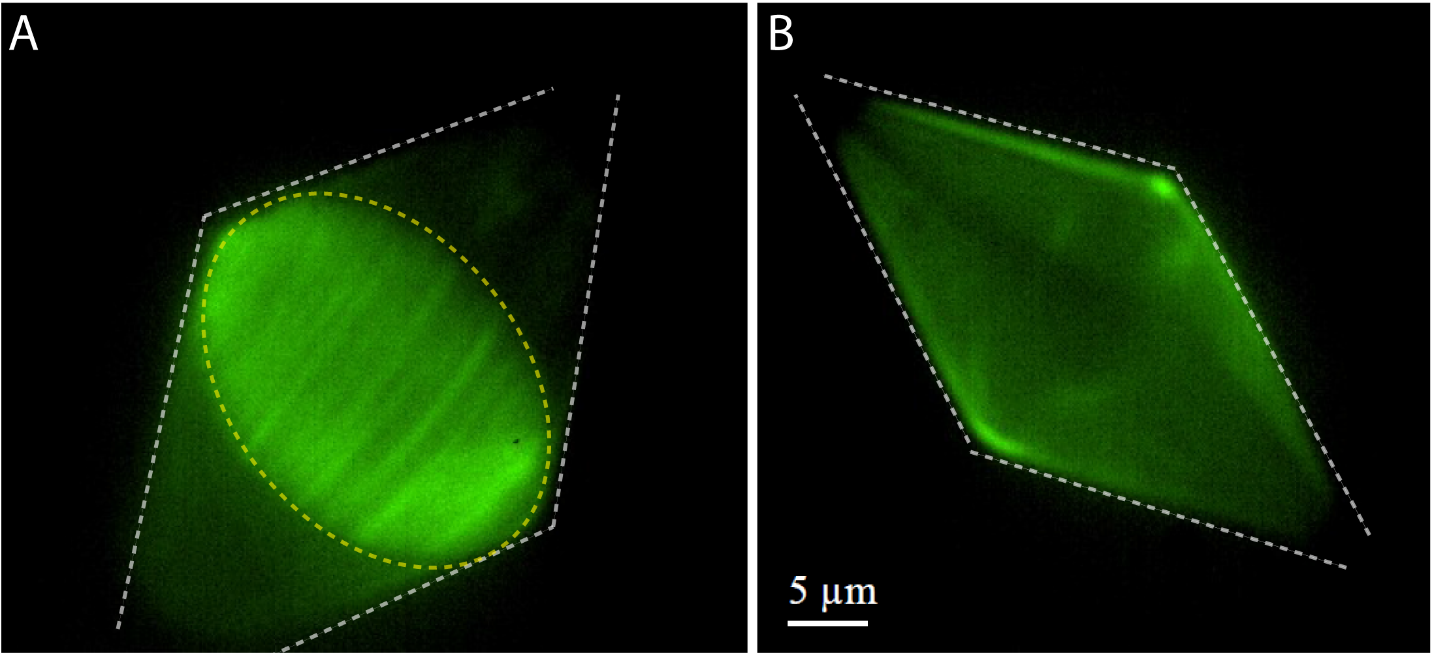


**Figure S1 - Ice crystals in GFP-tagged AFPIII after protein solution exchange.** A– The crystal was prepared in a high concentration of AFPIII-GFP, and then the excess AFPIII-GFP solution was removed. Note - the fluorescent oval core from AFPIII-GFP bound during initial ice formation. B – The initial ice seed was prepared in the absence of AFPIII-GFP, and then an AFPIII-GFP solution was introduced. The temperature was decreased and the crystal was shaped as a hexagonal bipyramid. After a few minutes the AFPIII-GFP solution was removed. This crystal has fluorescence on the surface only and none in the core.


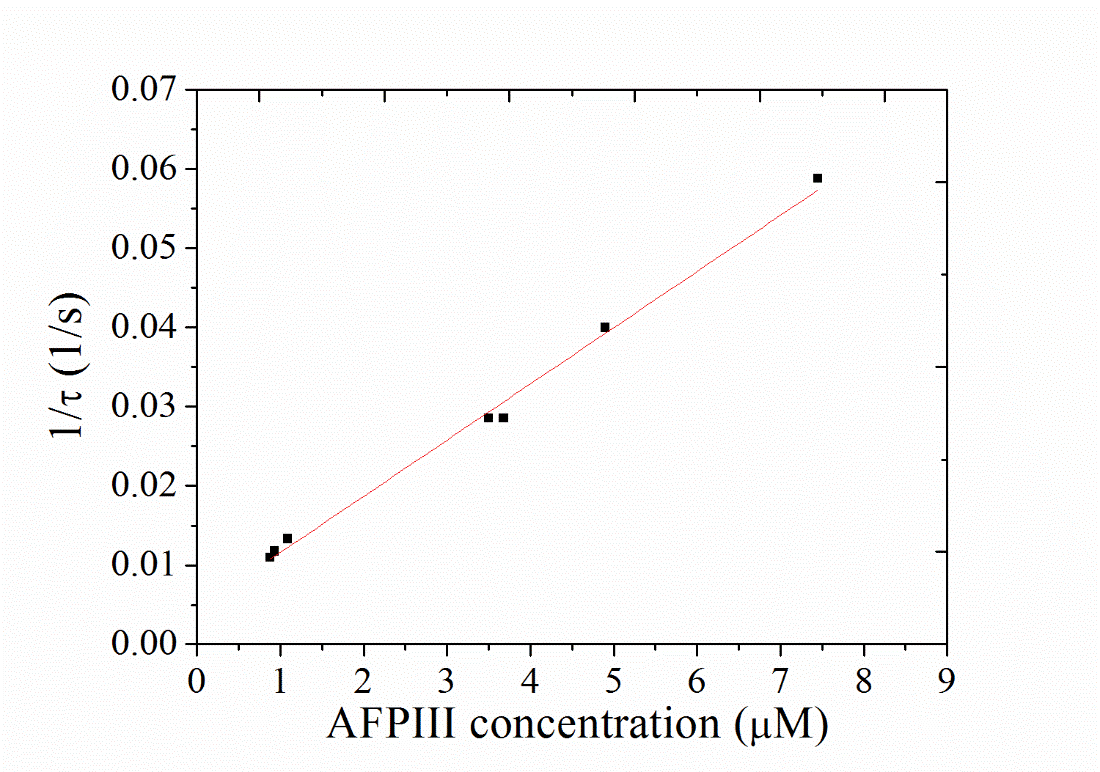


**Figure S2 – Calculation of K_on_.** The solution concentration of AFPIII-GFP was plotted against the inverted time constant τ, obtained by fitting the accumulation curves using the formula$I=I_{MAX}\left(1-e^{-\left(\frac{t}{\tau}\right)}\right)$. Each data point represents one crystal. Assuming that
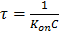
 , K_on_ was calculated to be 0.008 ± 0.001 µM^-1^ s ^-1^.


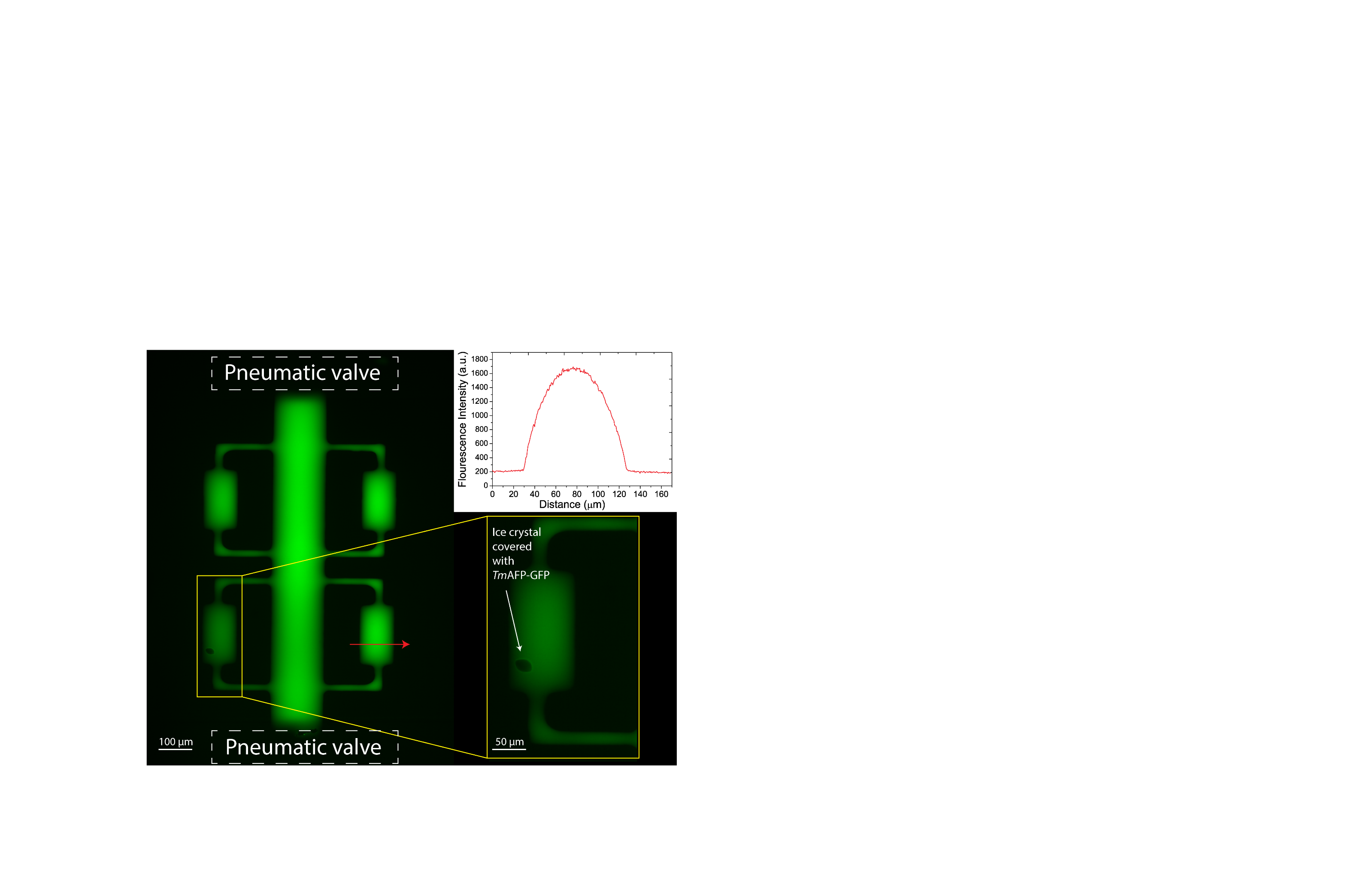


**Figure S3 – Design of the microfluidic channel.** The small cells above were designed to accommodate the formed ice crystals, and to prevent the melting of these crystals during solution exchange. In order to close the flow channels using the pneumatic valves, a round flow channel was used. We measured the fluorescent intensity perpendicular to the bottom right cell (marked by a red arrow). The above graph shows that the channel is indeed round shaped.

**S4 – movie - Accumulation of *Tm*AFP the basal plane** over time.


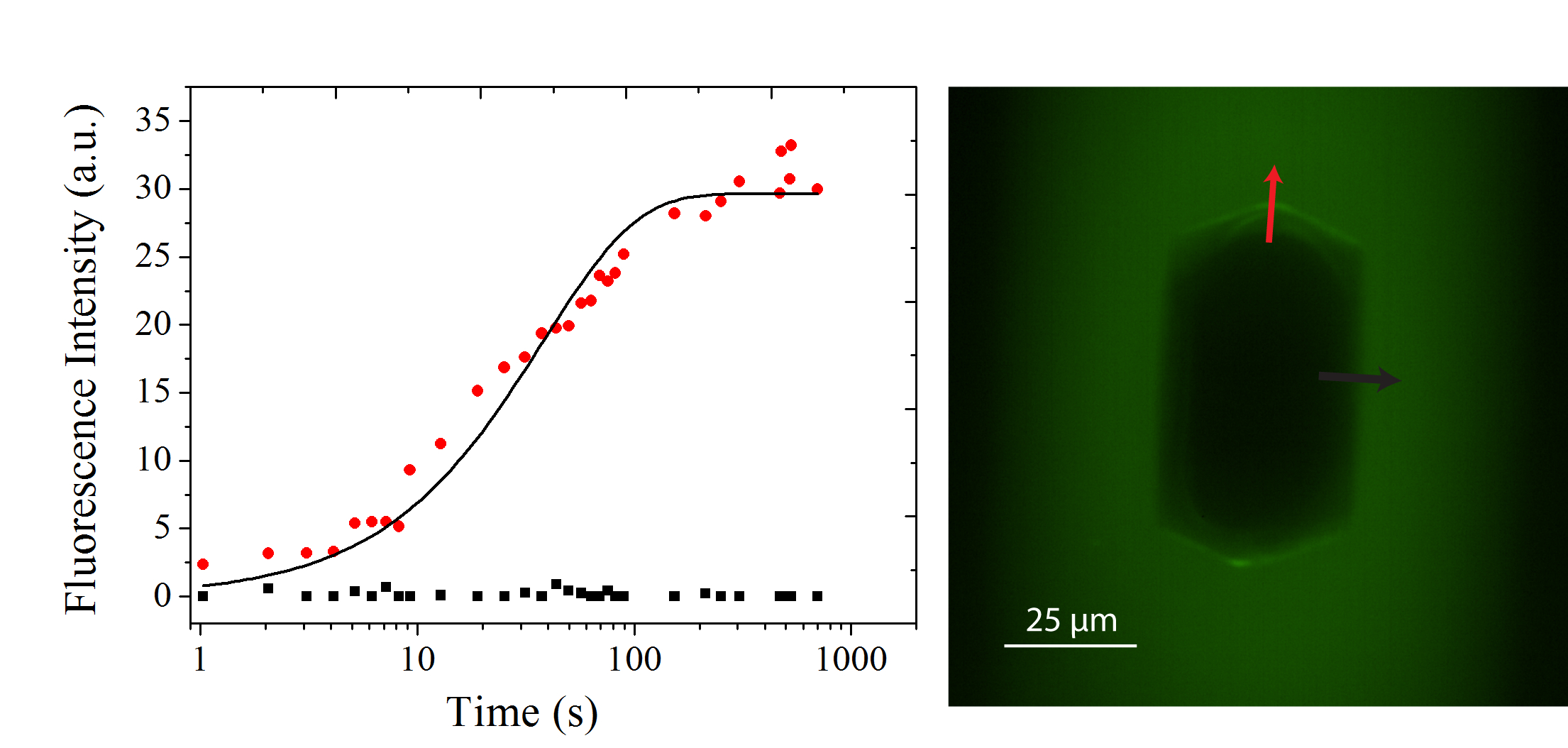


**Figure S4 - Accumulation of AFPIII on different ice crystal planes**. Right panel – ice crystal adsorbing AFPIII-GFP with the relevant crystal axes shown (red arrow – measurement on the prism plane, black arrow – measurement on the basal plane). Left panel – measurement of the fluorescence intensity over time on the stated crystal planes. Black squares – basal plane, Red circles – prism plane. The data was fitted using $I=I_{MAX}\left(1-e^{-\left(\frac{t}{\tau}\right)}\right)$ - black curve.
